# Withanolides-Induced Breast Cancer Cell Death Is Correlated with Their Ability to Inhibit Heat Protein 90

**DOI:** 10.1371/journal.pone.0037764

**Published:** 2012-05-31

**Authors:** Hui-Chun Wang, Yi-Ling Tsai, Yang-Chang Wu, Fang-Rong Chang, Mei-Hsin Liu, Wen-Ying Chen, Chin-Chung Wu

**Affiliations:** 1 Graduate Institute of Natural Products, Kaohsiung Medical University, Kaohsiung, Taiwan; 2 School of Chinese Medicine, College of Chinese Medicine, China Medical University, Taichung, Taiwan; 3 Cancer Center, Kaohsiung Medical University Hospital, Kaohsiung, Taiwan; 4 Basic Medical Science Education Center, Fooyin University, Kaohsiung, Taiwan; Wayne State University, United States of America

## Abstract

Withanolides are a large group of steroidal lactones found in Solanaceae plants that exhibit potential anticancer activities. We have previously demonstrated that a withanolide, tubocapsenolide A, induced cycle arrest and apoptosis in human breast cancer cells, which was associated with the inhibition of heat shock protein 90 (Hsp90). To investigate whether other withanolides are also capable of inhibiting Hsp90 and to analyze the structure-activity relationships, nine withanolides with different structural properties were tested in human breast cancer cells MDA-MB-231 and MCF-7 in the present study. Our data show that the 2,3-unsaturated double bond-containing withanolides inhibited Hsp90 function, as evidenced by selective depletion of Hsp90 client proteins and induction of Hsp70. The inhibitory effect of the withanolides on Hsp90 chaperone activity was further confirmed using *in vivo* heat shock luciferase activity recovery assays. Importantly, Hsp90 inhibition by the withanolides was correlated with their ability to induce cancer cell death. In addition, the withanolides reduced constitutive NF-κB activation by depleting IκB kinase complex (IKK) through inhibition of Hsp90. In estrogen receptor (ER)-positive MCF-7 cells, the withanolides also reduced the expression of ER, and this may be partly due to Hsp90 inhibition. Taken together, our results suggest that Hsp90 inhibition is a general feature of cytotoxic withanolides and plays an important role in their anticancer activity.

## Introduction

Withanolides are a group of naturally occurring steroidal lactones which are found in certain genera of the Solanaceae family. Some withanolide-containing plants have been known as folk remedies for centuries. For example, *Withania somnifera* contains a major withanolide- withaferin A, has been traditionally used in India for treatment of arthritis and other musculoskeletal conditions, and as a general tonic [1]. Pharmacological studies have revealed that besides anti-inflammatory activity, withanolides possess immunoregulatory, anti-tumor, anti-angiogenic, anti-invasive, and chemopreventive effects [2]. In addition, there are over 350 withanolides isolated and identified to date [3]; the diverse structural analogs provide a great opportunity for the study of structure-activity relationships, target identification, and lead optimization. Therefore, withanolides represent promising lead compounds for development of new anticancer drugs.

In our previous study, we have found that tubocapsenolide A, a novel withanolide from a native Taiwanese plant *Tubocapsicum anomalum* (Franchet and Savatier) Makino (Solanaceae), exhibited significant cytotoxicity against a panel of cancer cell lines [4]. Further study on the mechanism of action revealed that tubocapsenolide A inhibited the function of heat shock protein 90 (Hsp90) through thiol oxidation, and thus caused the depletion of Hsp90 client proteins, including Akt, CDK4, cyclin D, and mutant P53 [5]. Because Hsp90 client proteins play critical roles in the regulation of cell proliferation, survival, and apoptosis, it is suggested that tubocapsenolide A exerted its anticancer effect by inhibiting Hsp90. Subsequent to our study, Yu et al. reported that another withanolide withaferin A also has the same inhibitory effect on Hsp90 [6]. More recently, by using molecular docking approach, Grover et al. predicted that WA has the potential to inhibit the association of Hsp90 to its co-chaperone Cdc37 by disrupting the stability of attachment of Hsp90 to Cdc37 [7]. Therefore, we wondered whether the Hsp90-inhibitory effect is a general characteristic of withanolides and contributes to their anticancer effect. In the present study, we compared nine withanolides with different structural features for their cytotoxicity and Hsp90-inhibitory activity, and found a good correlation between these two effects. In addition, structure activity analysis revealed a critical requirement of the α, β-unsaturated ketone unit in the ring A of withanolides for the inhibitory effect on Hsp90.

## Materials and Methods

### Materials

Tubocapsenolide A, tubocapsenolide B, tubocapsanolide C, tubocapsanolide E, and anomanolide A were isolated from *Tubocapsicum anomalum* as described previously [4]. 4β-Hydroxywithanolide, withanolide E, and peruvianolide H were isolated from *Physalis peruviana* [8]. Withaferin A was purchased from ChromaDex (Irvine, CA). The purities of the withanolides used in the study are over 98% determined by high pressure liquid chromatography. The withanolides were dissolved in DMSO, and the final concentration of DMSO in culture medium was 0.1%. Anti-Poly(ADP-ribose)polymerase, anti-caspase-3, anti-Raf-1, anti-CDK4 antibodies were from Santa Cruz Biotechnology Inc. (Santa Cruz, CA). Anti-Akt, anti-IKKα, anti-IKKβ, anti-IκB, anti-phospho-IκB, anti-NF-κB, and anti-phospho-NF-κB antibodies were from Cell Signaling Technology (Beverly, MA). Anti-Hsp90 and anti-Hsp70 antibodies were from Stressgen Biotechnologies (San Diego, CA). ONE-Glo™ luciferase assay system was purchased from Promega (Madison, WI). Geldanamycin, N-acetylcysteine, 3-(4,5-dimethyl-thiazol-2-yl)-2,5-diphenyl tetrazolium bromide (MTT), and all other chemicals were obtained from Sigma Chemical Co (St Louis, MO).

### Cell culture

MDA-MB-231 and MCF-7 human breast cancer cells were obtained from American Tissue Culture Collection. Cells were incubated at 37°C in a humidified atmosphere containing 5% CO_2_ in RPMI 1640 medium, supplemented with 10% fetal bovine serum, penicillin (100 IU/ml) and streptomycin (100 µg/ml). The cells were harvested by trypsinization and plated 24 h before treatment with the test drugs.

### Evaluation of cell viability

1×10^4^ cells were plated into each well of a 96-well plate and treated with the various concentrations of drugs for different indicated times. At the end of each time point, 100 µl of MTT solution (0.5 mg/ml) was added to each well. Cells were then incubated at 37°C for 1 h. The MTT crystals in each well were solubilized with 100 µl of DMSO. Absorbance was read at 550 nm.

### Western blot assay

Following exposure to various drugs, cancer cells were washed with PBS and lysed for 10 min at 4°C in a lysis buffer (50 mM Tris-HCl, 150 mM NaCl, 1 mM EDTA, 1 mM EGTA, 1% Triton X-100, 20 µg/ml leupeptin, 2 mM sodium orthovanadate, 1 mM phenylmethylsulfonyl fluoride, 5 mM sodium fluoride, and 5,000 units/ml aprotinin). Equal proteins were separated by SDS-PAGE and transferred to a nitrocellulose membrane, followed by visualization using the enhanced chemiluminescence (ECL) reagent (Amersham Pharmacia Biotech). For analysis of disulfide-linked Hsp90 by non-reducing SDS-PAGE, drug-treated cells were washed with PBS at the indicated periods and then incubated with iodoacetamide (40 mM) for 5 min to prevent thiol-disulfide exchange and post-lysis oxidation of free cysteines [9]. Moreover, samples were diluted in SDS-sample buffer without reducing agents before loading onto SDS-polyacrylamide gels.

### Annexin V/propridium iodide (PI) staining

Annexin V/PI staining was performed as previously described [10]. Cancer cells (2×10^5^ cells) were seeded onto each well of a 6-well plate and treated with DMSO or test drugs for 48 h. Treated cells were harvested by trypsinization and stained with annexin V and PI for 15 min at room temperature in the dark according to the manufacturer's instructions (BD Pharmingen). Apoptotic cells were quantified by flow cytometry (Beckman Coulter, EPICS XL-MCL). Stained cell populations were defined as: lower left quadrant, living cells (annexin V^−^/PI^−^); lower right quadrant, early apoptotic cells (annexin V^+^/PI^−^); upper right quadrant, late apoptotic cells (annexin V^+^/PI^+^); upper left quadrant, primary necrotic cells (annexin V^−^/PI^+^).

### In vivo Hsp90 chaperone activity assay

The measurement of Hsp90 chaperone activity in intact cells was carried out by the methods of Schneider et al. [11] and Hernández et al. [12] with slight modifications. MDA-MB-231 cells were transfected with a pGL2-sv40-luciferase plasmid. The luciferase-expressed cells were treated with DMSO or test drugs at 37°C for 30 min, and then transferred to 42°C for another 15 min. After heat shock treatment, the cells were left to recover at 37°C for 10 or 60 min. Subsequently, luciferase activity in the cells was determined by One-Glo™ luciferase assay (Promega, Madison, WI) according to the manufacturer's protocol.

### shRNA knockdown of Hsp70 in MDA-MB-231 cells

shRNA plasmid against Hsp70 (clone TRCN0000005945) or control plasmid (clone TRCN0000072223) were obtained from the National RNAi Core Facility located at the Institute of Molecular Biology/Genomic Research Center, Academia Sinica, Taiwan. To knockdown Hsp70 expression in MDA-MB-231 cells, shRNA plasmid against Hsp70 or control plasmid was introduced into the cells using Lipofectamine 2000 (Invitrogen) according to the manufacturer's protocol. Forty-eight hours following transfection, the cells were treated with the withanolides.

### Statistics

Data are presented as means ± S.E.M., and comparisons were made using Student's *t* test or one-way ANOVA. A probability of 0.05 or less was considered statistically significant.

## Results

### Structure-cytotoxicity relationships of withanolides in MDA-MB-231 cells

**Figure 1:**
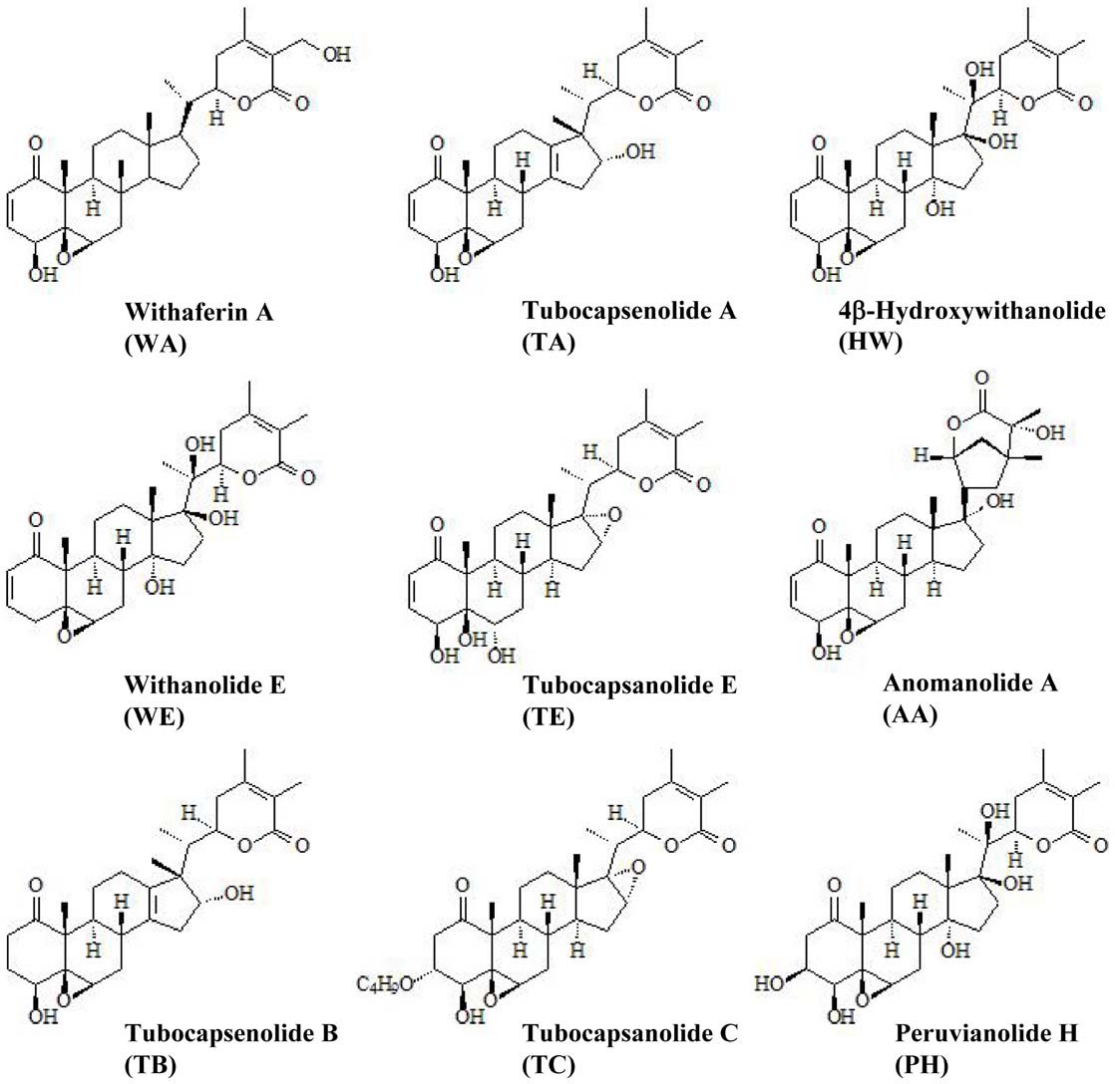
Chemical structures of the withanolide compounds.

**Figure 2:**
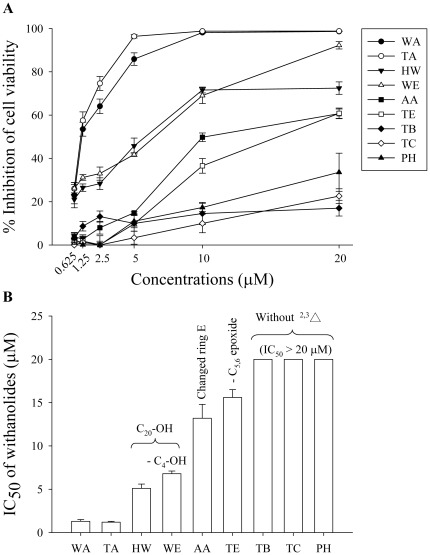
Effect of the withanolides on the viability of MDA-MB-231 cells. (A) MDA-MB-231 cells were treated with the indicated concentrations of various withanolide compounds for 48 h, and cell viability was determined by the MTT assay. (B) The IC_50_ values of the withanolides on the viability of MDA-MB-231 cells. Results are presented as means ± S.E.M. (n = 3).

Nine withanolides, including withaferin A (WA), tubocapsenolide A (TA), 4β-hydroxywithanolide (HW), withanolide E (WE), tubocapsanolide E (TE), anomanolide A (AA), tubocapsenolide B (TB), tubocapsanolide C (TC), and peruvianolide H (PH) (Fig. 1), were tested in the human breast cancer MDA-MB-231 cells for their cytotoxicity using the MTT assay. Fig. 2 shows that these withanolides reduced cell viability with different potency. The analysis of the structure-cytotoxicity relationships reveals that the α, β-unsaturated ketone in ring A is essential for the cytotoxicity of withanolides, since TB, TC, and PH, which lack the 2,3-unsaturated double bond, lost cytotoxic activity in MDA-MB-231 cells. Among the 2,3-unsaturated double bond-containing withanolides, WA and TA are the most potent cytotoxic compounds, and both have an additional 4β-hydroxy group and a 5β,6β-epoxide. In contrast, compounds lacking the 4β-hydroxy group (i.e., WE) or the 5β,6β-epoxide (i.e., TE) significantly reduced their cytotoxicity. These results suggest either or both of the two substitutions can enhance the cytotoxicity of withanolides. On the other hand, the addition of a hydroxyl group to C20 (i.e., HW and WE) reduces the cytotoxic effect of withanolides.

### Withanolides induce apoptosis in MDA-MB-231 cells

**Figure 3:**
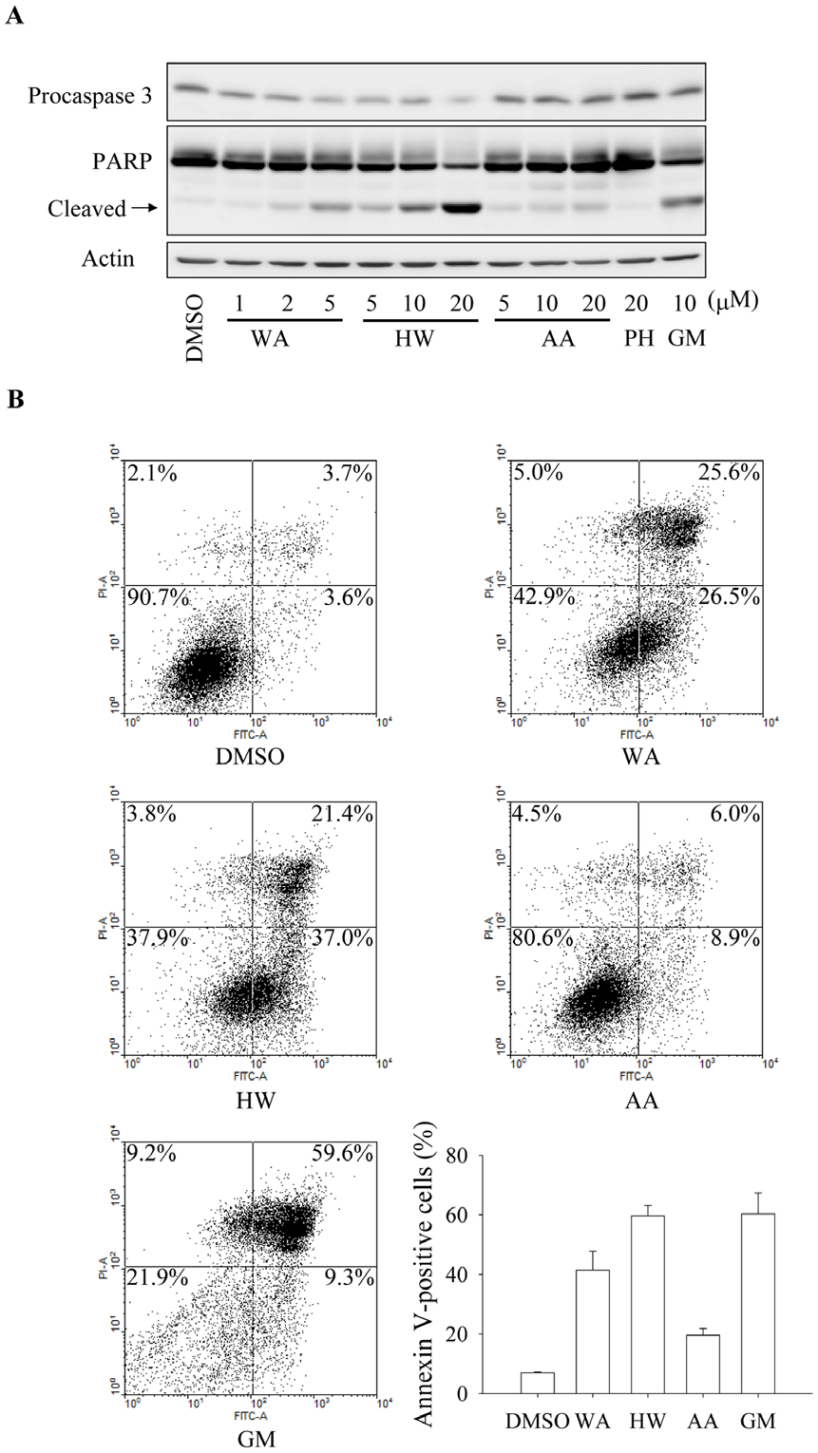
Withanolides induce apoptosis in MDA-MB-231 cells. (A) Cells were treated with the indicated concentrations of WA, HW, AA, and geldanamycin (GM) for 48 h. After treatment, cells were harvested and analyzed for PARP and caspase-3 by Western blot. (B) Cells were treated with WA (5 µM), HW (20 µM), AA (20 µM), or GM (10 µM) for 48 h. Harvested cells were then stained with annexin V-FITC and PI and analyzed by flow cytometry. Percentages of annexin V-positive cells were calculated by combining annexin V^+^/PI^−^ (lower right quadrants) and annexin V^+^/PI^+^ (upper right quadrants) cells. Results are presented as means ± S.E.M. (n = 3).

WA, HW, AA, and PH were chosen for the further studies based on their different chemical characteristics and cytotoxicity. In addition, the well-known Hsp90 inhibitor geldanamycin (GM) was also studied in parallel with the withanolides. We investigated if the withanolides induce apoptotic death in MDA-MB-231 cells. The activation of caspase-3, which is an important effector caspase, has been used as a indicator of apoptosis and, this process can be determined by a decrease in pro-enzyme levels using Western blot analysis. Figure 3a showed that the protein levels of procaspase-3 were decreased in MDA-MB-231 cells after treatment of WA (5 µM), HW (20 µM), AA (20 µM), or GM (10 µM) for 48 h. The activation of caspase-3 by the withanolides and GM was further confirmed by detecting the degradation of PARP, which is a DNA repair enzyme and undergo cleavage by caspase-3 during apoptosis. In contrast to other withanolides, PH induced neither caspase-3 activation nor PARP cleavage.

In order to quantify withanolide-induced apoptosis, we also performed flow cytometric analysis using annexin V-FITC/PI double staining. Annexin V has been widely used to detect the exposed phosphatidylserine on cell surface during apoptosis. When combined with PI, which stains cells that have lost membrane integrity, this approach allows a further distinction of early apoptotic (annexin V^+^/PI^−^), late apoptotic/necrotic (annexin V^+^/PI^+^) cells, and primary necrotic cells (annexin V^−^/PI^+^). Figure 3b shows that treatment of MDA-MB-231 cells with WA (5 µM), HW (20 µM), AA (20 µM), or GM (10 µM) for 48 h led to significant increases of both early and late apoptotic cells.

### Withanolides induce degradation of Hsp90 client proteins and induction of Hsp70

**Figure 4:**
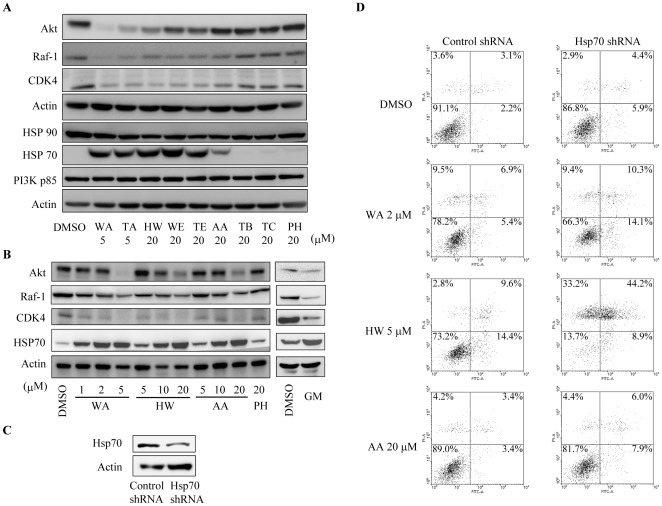
Withanolides induce degradation of Hsp90 client proteins and induction of Hsp70 in MDA-MB-231 cells. (A) Cells were treated with the indicated concentrations of various withanolide compounds for 12 h. After treatment, cells were harvested and analyzed for the Hsp90 client proteins (Raf-1, CDK4, and Akt), a non-Hsp90 client protein p85, and Hsp70 by Western blot. (B) Concentration-dependent effect of WA, HW, and AA on the Hsp90 client proteins and Hsp70. GM (10 µM) was used as a positive control. (C, D) Knockdown of Hsp70 enhances the pro-apoptotic effect of the withanolides. MDA-MB-231 cells were transfected with shRNA plasmid against Hsp70 or control plasmid for 48 h. The knockdown efficiency of shRNA for Hsp70 was confirmed by Western blot (C). The transfected cells were treated with WA (2 µM), HW (5 µM), or AA (20 µM) for another 48 h for determining apoptosis (D).

We next determined the effect of withanolides on protein levels of the Hsp90 client proteins Akt, Raf-1, and CDK4 in MDA-MB-231 cells. As shown in Fig. 4A and B, at the concentration of 5 µM, both WA and TA treatments markedly reduced Akt, Raf-1, and CDK4 levels. At a higher concentration (20 µM), HW, WE, TE, and AA also caused significant decreases in Raf-1 and CDK4 levels and, to a less extent, Akt levels. The effect of the withanolides on Hsp90 client proteins was accompanied by an increase in Hsp70 expression. In contrast, the non-Hsp90 client protein PI3K p85 subunit was not affected. Importantly, TB, TC, and PH, which are weakly cytotoxic withanolides, did not reduce the amounts of the Hsp90 client proteins and did not induce Hsp70 expression in cancer cells.

Hsp70 is known as an anti-apoptotic protein, and previous studies have shown that induction of Hsp70 following Hsp90 inhibition attenuates the cytotoxic effect of Hsp90 inhibitors [13], [14]. In order to determine the role of Hsp70 induction on withanolide-induced apoptosis, we used shRNA to reduce Hsp70 levels in MDA-MB-231 cells. The capacity of the shRNA against Hsp70 was confirmed using Western blot (Fig. 4 C). As shown in Fig. 4D, Hsp70 knockdown enhanced the pro-apoptotic effect of WA, HW, and AA. This result suggests that like other Hsp90 inhibitors, up-regulation of Hsp70 also plays a protective mechanism against withanolide-induced apoptosis.

### Withanolides inhibit the chaperone activity of Hsp90

**Figure 5:**
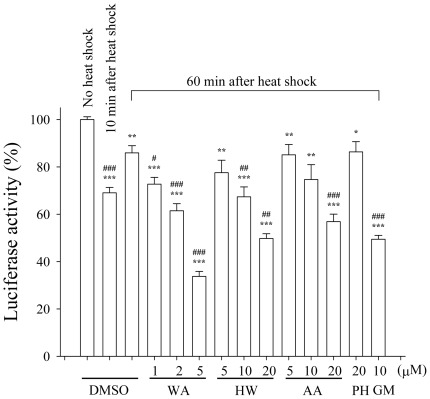
Effect of heat shock on luciferase expressed in vivo in the presence of withanolides. MDA-MB-231 cells expressed luciferase were pretreated DMSO or withanolides in 96-well plates for 30 min before incubation at 42°C for 15 min. After heat shock treatment, cells were left to recover at 37°C for 10 or 60 min. Luciferase activity was assayed on whole-cell lysates. Results are presented as means ± S.E.M. (n = 3). *P<0.05, **P<0.01, **P<0.001 as compared with the no heat shock group. ^#^ *P*<0.05, ^##^ *P*<0.01, ^###^ *P*<0.001 as compared with the DMSO group which was recovered for 60 min after heat shock.

In order to investigate if withanolides-induced degradations of Akt, Raf-1, and CDK4 were due to inhibition of the chaperone activity of Hsp90, an *in vivo* heat shock luciferase activity recovery assay was used. It has been reported that firefly luciferase expressed in mammalian cells denatures at high temperatures but refolds upon temperature downshift in an Hsp90-dependent manner [11]. In the present study, 293T cells transfected with firefly luciferase-expressing plasmid was heat-stressed at 42°C for 15 min. The cells were then shifted to 37°C for 10 min and 60 min to recover the luciferase activity. As shown in Fig. 5, after 10 min and 60 min recovery, the luciferase activity reaching 69% and 86% of control activity, respectively. In the presence of geldanamycin (10 µM), luciferase activity was only 49% of control levels after 60 min recovery at 37°C, indicating that Hsp90 involves in the refolding processes. Similar to geldanamycin, WA (1–5 µM), HW (5–20 µM), and AA (5–20 µM) inhibited luciferase refolding in a concentration-dependent manner. In contrast, PH did not affect the recovery of luciferase activity.

### Withanolides-induced cell death and Hsp90 inhibition are prevented by N-acetylcysteine

**Figure 6:**
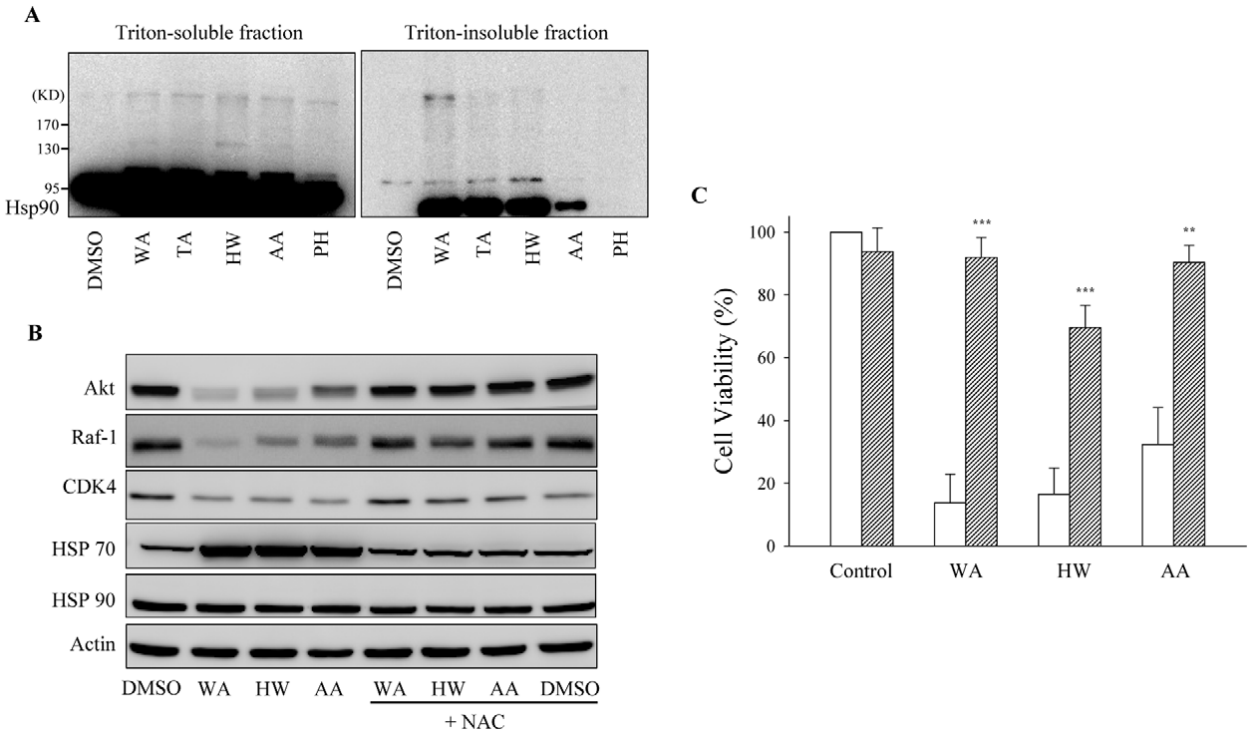
Withanolides cause thiol oxidation and aggregation of Hsp90 in MDA-MB-231 cells. (A) Cells were treated with WA (5 µM), TA (5 µM), HW (20 µM), AA (20 µM) or PH (20 µM) for 12 h. The Triton-insoluble fractions of cell lysates were prepared as described under “Experimental Procedures.” The protein levels of the disulfide-linked-Hsp 90 were analyzed by nonreducing SDS-PAGE. (B, C) The thiol-reducing agent NAC prevents the degradation of Hsp90 client proteins and cell death caused by withanolides. MDA-MB-231 cells were pretreated with or without NAC (2.5 mM) for 1 h before challenged with withanolides for 12 h (B) or 48 h (C). The protein levels of heat shock proteins and client proteins were analyzed by western blot (B). The viability of MDA-MB-231 cells was determined by the MTT assay (C). Results are presented as means ± S.E.M. (n = 3). **P<0.01, ***P<0.001 as compared with each group without NAC pretreated.

In the previous studies, TA and WA have been reported to induce thiol oxidation and aggregation of Hsp90 by promoting the formation of intermolecular disulfide bonds [5], [6]. In the present work, we found that when MDA-MB-231 cells were treated with WA, TA, HW or AA, disulfide-linked Hsp90 with higher molecular weight appeared in both the Triton-soluble fraction and the Triton-insoluble fraction, and migrated slower than the native Hsp90 in non-reducing SDS-PAGE (Fig. 6A). These results suggested that Hsp90 was oxidative-damaged and formed insoluble aggregates in cancer cells treated with the withanolides. In contrast, the less-cytotoxic withanolide PH, had no effect on the oxidation and aggregation of Hsp90.

We next used an antioxidant N-acetylcysteine (NAC), which contains a reduced sulfhydryl group, to prevent the oxidation of Hsp90. In the presence of NAC, the effects of WA, HW, and AA on Hsp90 client proteins and Hsp70 induction were abolished (Fig. 6B). Furthermore, cell death induced by these withanolides was also markedly prevented (Fig. 6C).

### Effect of withanolides and geldanamycin on IKK and NF-κB

**Figure 7:**
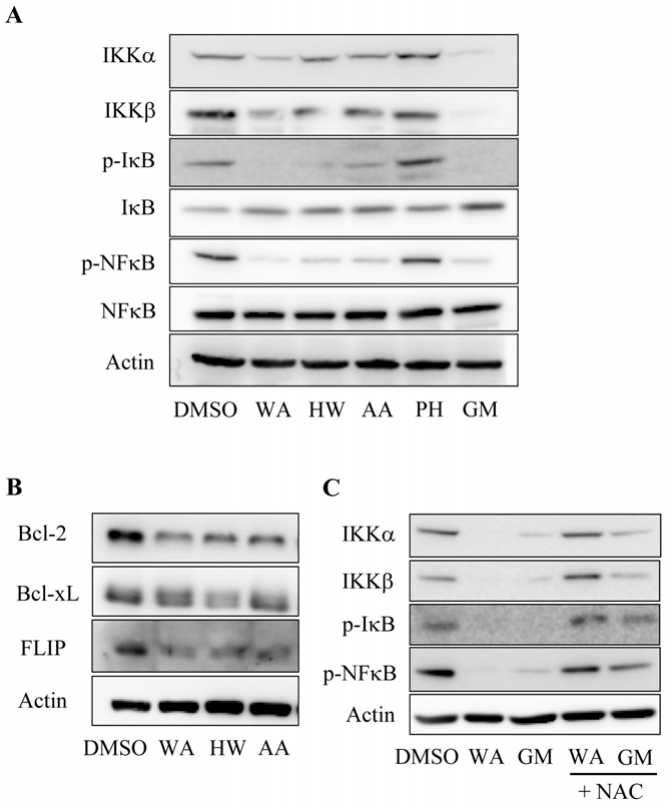
Effect of withanolide on the IKK/NF-κB pathway. (A) Withanolides induce IKK degradation and inhibit NF-κB activation. MDA-MB-231 cells were treated with WA (5 µM), HW (20 µM), AA (20 µM), PH (20 µM) or GM (10 µM) for 12 h. After treatment, cells were harvested and analyzed for the proteins involved in the NF-κB pathway by Western blotting. (B) Withanolides decrease anti-apoptotic proteins that are regulated by NF-κB. Cells were treated with withanolides for 48 h. After treatment, cells were harvested and analyzed for Bcl-2, Bcl-xL, and c-FLIP by Western blotting. (C) NAC prevents IKK degradation and NF-κB inhibition by WA. Cells were treated with WA (5 µM) or GM (10 µM) in the absence or presence of NAC (2.5 mM) for 12 h. After treatment, cells were harvested and subjected to Western blotting.

IκB kinase (IKK), a key regulator in the NF-κB pathway, has been reported to be a Hsp90 client protein [15]. In addition, the NF-κB pathway is constitutively active in estrogen receptor (ER)-negative breast cancer cells and contributes to their hormone-independent growth [16]. Therefore, we wanted to investigate the effect of withanolides on IKK and NF-κB. Fig. 7A shows that treatment of MDA-MB-231 cells with WA, HW or AA led to a decrease in both IKKα and IKKβ. The rank order of potency was WA>HW>AA. Geldanamycin treatment also induced IKK depletion. Withanolides- and geldanamycin- induced IKK depletion was accompanied by IκB de-phosphorylation and IκB accumulation and, resulted in inhibition of NF-κB activation as evidenced by the decrease in Ser536 phosphorylation. The inhibition of the NF-κB pathway by withanolides was further supported by that NF-κB-regulated gene products Bcl-2, Bcl-xL, and c-FLIP [17], [18] were reduced by WA, HW, and AA. PH, which was inactive in inhibiting Hsp90, did not affect IKK levels and failed to inhibit NF-κB activation (Fig. 7B).

In addition, NAC pretreatment completely prevented the effect of withanolides on IKK and NF-κB. In contrast, NAC only partially reversed the effect of geldanamycin (Fig. 7C).

### Effect of the withanolides on ER-positive human breast cancer cells

**Figure 8:**
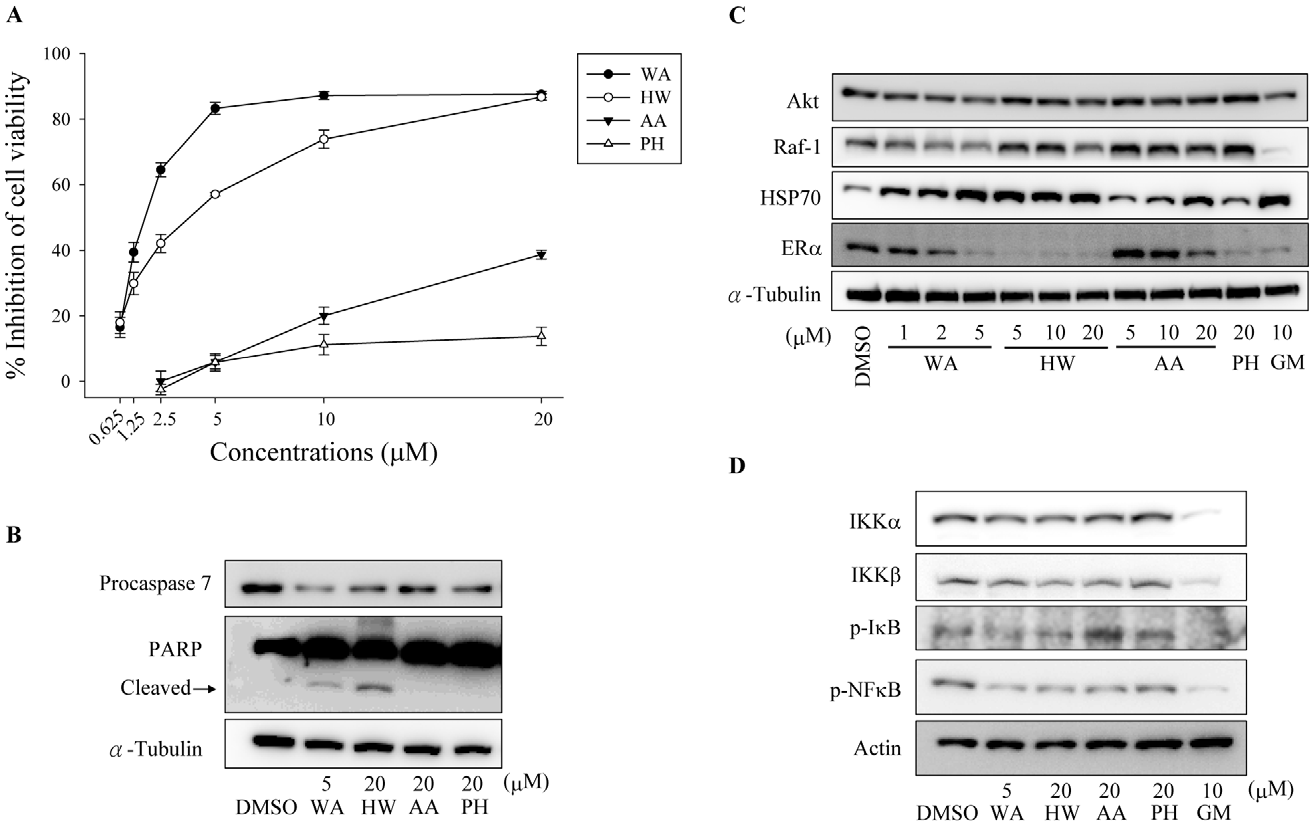
Effect of the withanolides on ER-positive human breast cancer cells. (A) Withanolides reduce the viability of MCF-7 cells. MCF-7 cells were treated with the indicated concentrations of WA, HW, AA, or PH for 48 h, and cell viability was determined by the MTT assay. (B) Withanolides induce caspase-7 activation and PARP cleavage. MCF-7 cells were treated with the withanolides for 48 h. After treatment, cells were harvested and analyzed for caspase-7 and PARP by Western blot. (C) Withanolides induce degradation of Hsp90 client proteins and induction of Hsp70. MCF-7 cells were treated with the withanolides or GM for 12 h. Levels of Hsp90 client proteins and Hsp70 were determined by Western blot. (D) Withanolides induce IKK degradation and inhibit NF-κB activation. MCF-7 cells were treated with the withanolides or GM for 12 h. After treatment, cells were harvested and analyzed for the proteins involved in the NF-κB pathway by Western blot.

In order to confirm the generality of the Hsp90-inhibitory effect of the withanolides, another human breast cancer cell line MCF-7, which expresses ER, was treated with the compounds. As shown in Fig. 8A, the withanolides reduced the viability of MCF-7 cells, with potency in the order WA>HW>AA>PH. WA and HW also induced apoptotic death in MCF-7 cells as evidenced by a decrease of procaspase-7 and the cleavage of PARP (Fig. 8B). Furthermore, in correlation with their cytotoxicity, the withanolides reduced the levels of Hsp90-client proteins (Akt and Raf-1) and induced Hsp70 overexpression in MCF-7 cells (Fig. 8C). In addition, the withanolides reduced the expression of ER in MCF-7 cells (Fig. 8C), but the rank order of potency was HW≧WA>PH>AA.

As observed in MDA-MB-231 cells, the treatment of MCF-7 cells with the withanolides also resulted in a decrease in the levels of both IKKα and IKKβ, with the consequent decrease in the phosphorylation of IκB and NF-κB (Fig. 8D).

## Discussion

There are several mechanisms of action proposed for withanolides, including NF-κB inhibition, PAR-4 induction, actin microfilament and/or vimentin intermediate filament aggregation, ROS formation, proteasome inhibition and Hsp90 inhibition [5], [19]–[24]; however, it remains unclear which of them is a common property and contributes mainly to the anticancer activities of withanolides. In this work, we found that a panel of withanolides inhibited Hsp90 function in human breast cancer cells, as measured by depletion of client proteins and by chaperone activity, to different extents. Furthermore, there is a good correlation between Hsp90 inhibition and the cytotoxic effect caused by the withanolides. These results support that Hsp90 inhibition is an important mechanism of anticancer activity of withanolides.

The Hsp90-inhibitory effect of withanolides was apparent due to induction of oxidative insults of Hsp90 as shown in the previous reports [5], [6], because withanolides that inhibited Hsp90 functions also induced Hsp90 oxidation and aggregation, and the thiol antioxidant NAC prevented both oxidative insults and function loss of Hsp90 caused by the withanolides. Structure-activity analysis of the withanolides in the present study indicates that the α, β-unsaturated ketone in ring A is critical for inducing Hsp90 oxidative damage. α, β-Unsaturated ketones are strong electrophilics which can react with protein cysteine residues by Michael addition that results in thiol alkylation and/or thiol oxidation [25]. Besides the α, β-unsaturated ketone, the epoxide in ring B and the conjugated ketone in the lactone ring of withanolides are also electrophilic moieties [26]. However, the electrophilicity of the conjugated ketone in the lactone ring is much lower than that of α, β-unsaturated ketone, since a methyl substitution at the β-position of the former prevents nucleophilic attack. As a result, the withanolides containing only a conjugated ketone in the lactone ring (eg. PH) had no or little effect on Hsp90 oxidation and inhibition.

Several withanolides, including WA, have been reported to inhibit NF-κB activation in tumor necrosis factor (TNF)-stimulated cells [19], [27], [28]. Although the mechanisms involved in NF-κB inhibition by withanolides are not fully understood, previous studies have shown that withanolides can inhibit IκB phosphorylation and degradation with consequent reduction of NF-κB in the nucleus [27]. The IκB kinase complex contains two kinase subunits, IKKα and IKKβ is the upstream kinase responsible for IκB phosphorylation, which leads to proteasome-dependent degradation of IκB. Both IKKα and IKKβ have been suggested as Hsp90 client proteins, since Hsp90 inhibition leads to the degradation of IKKs [15], [29], [30]. In the present study, we show for the first time that withanolides caused both IKKα and IKKβ depletion in human breast cancer cells; moreover, this effect was correlated with their Hsp90-inhibitory activities. The depletion of IKKs by withanolides was accompanied with reduced both phosphorylation and degradation of IκB, and led to inhibition of NF-κB activation, indicating that withanolides inhibit the NF-κB pathway by targeting IKKs.

NF-κB is constitutively active in most types of cancer and plays an important role in the development and progression, and contributes to the resistance of cancer cells to chemotherapy and radiotherapy [31], [32]. In human breast cancer cell lines or specimens, the NF-κB signaling is inversely related to estrogen receptor (ER) expression [16], [33]. The constitutive activation of NF-κB promotes the more invasive phenotype of ER-negative cancer cells via induction of anti-apoptotic and pro-metastatic genes, such as Bcl-2 and granulocyte macrophage-colony stimulating factor (GM-CSF) [34], [35]. Moreover, the inhibition of NF-κB signaling induces apoptosis in ER-negative breast cancer cells [36], [37]. These findings suggest that NF-κB is a potential target for the treatment of metastatic estrogen-independent breast cancer. In the present study, we show that withanolides inhibited the constitutive activation of NF-κB in MDA-MB-231, an ER-negative human breast cancer cell line, and reduced the anti-apoptotic proteins (Bcl-2, Bcl-xL, and c-FLIP) which are regulated by NF-κB. Given the importance of NF-κB in cancer cell survival, it is suggested that the inhibition of the IKK/NF-κB pathway may contribute to the anticancer effect of withanolides.

In contrast to MDA-MB-231 cells, the survival of ER-positive breast cancer cells, such as MCF-7, are less dependent on the IKK/NF-κB pathway [37]. Therefore, the inhibition of IKK/NF-κB by withanolides may not play a major role in their cytotoxicity against MCF-7. On the other hand, the withanolides exhibits potent inhibition of ER expression, an effect potentially lined to suppression of ER-dependent proliferation of breast cancer cells [38]. The reduced ER expression by withanolides may be, at least in part, due to inhibition of Hsp90, since ER is also a Hsp90 client protein [39], [40]. Of note, HW exhibits greater inhibitory effect on ER expression than that of WA suggesting that mechanisms other than Hsp90 inhibition may also contribute to its action on ER.

In conclusion, structure-activity analysis of a panel of withanolides reveals that 2,3-unsaturated double bond-containing withanolides exhibit potent inhibitory effect on Hsp90 chaperone activity. The inhibition of Hsp90 by the withanolides causes the depletion of several important signaling molecules involved in cell survival, including IKKs, and thus contributes to the anticancer effect of the withanolides.
